# Comparison of Radial Artery Occlusion Following Transradial Access for Percutaneous Coronary Intervention Using Sheath-based versus Sheathless Technique

**DOI:** 10.1038/s41598-018-30462-1

**Published:** 2018-08-13

**Authors:** Ala Mohsen, Musab Alqasrawi, Ghanshyam Palamaner Subash Shantha, Chris DeZorzi, Sidakpal Panaich

**Affiliations:** Department of Internal Medicine, University of Iowa, Iowa city, IA USA

## Abstract

We compared the risk of radial artery occlusion (RAO) in patients undergoing coronary intervention with introducer sheath (SG) or without introducer sheath (SLG). 1251 consecutive patients, from 2 tertiary care center in Pennsylvania, USA, undergoing percutaneous coronary interventions (PCI) between 2008–2013 formed the study cohort (SLG: 161 patients, SG: 1090 patients). Radial artery patency was assessed using plethysmography. The association between sheath use and RAO was assessed using unadjusted, adjusted and propensity macthed logistic regression analyses. Mean age: 65 years, men: 63%, diabetics: 37%. SG was associated with lower RAO at band removal [unadjusted (OR: 0.31, 95% CI: 0.21–0.46), adjusted (OR: 0.10, 95% CI: 0.05–0.20) and propensity matched (OR: 0.20, 95% CI: 0.13–0.32)], at 24 hours [unadjusted (OR: 0.20, 95% CI: 0.12–0.34), adjusted (OR: 0.12, 95% CI: 0.06–0.24) and propensity matched (OR: 0.13, 95% CI: 0.07–0.25)] and 30 days [unadjusted (OR: 0.28, 95% CI: 0.14–0.54), adjusted (OR: 0.22, 95% CI: 0.10–0.50) and propensity matched (OR: 0.18, 95% CI: 0.10–0.40)], compared to SLG. Sheath use during radial access for PCI is associated with less RAO. It is unclear if use of introducer sheath during radial access for PCI reduces incidence of RAO. In this prospective cohort study involving 1251 concecutive patients undergoing PCI via radial access between 2008–2013, we assessed the difference in incidence of RAO between the SG (n = 1090) and the SLG (n = 161 patients) groups. SG group experienced lower incidence of RAO at band removal, 24 hours and 30 days post PCI in the unadjusted, adjusted, and propensity matched analyses compared to the SLG group. In conclusion sheath use during radial access for PCI is associated with less RAO.

## Introduction

Transradial access (TRA) has increased steadily in utilization across the world since its original description^1–3^. For majority of percutaneous coronary intervention (PCI) procedures, a 5 or 6 french guide catheter provides sufficient lumen diameter to deploy required hardware. For a minority of interventional subsets, such as CTO, large burr size use for rotational atherectomy, and certain 2-stent technique bifurcation PCI, a larger than 6 french guide catheter may be needed. Introducer sheaths need to have an inner diameter that is at least as large as the outer diameter of the guide catheter, and hence an equivalent size introducer sheath is significantly larger compared to its corresponding guide catheter.

Post procedural loss of patency is the most common structural complcation of TRA with incidence varying from 2–10%^4^. Sheath to artery ratio has been shown to be a significant predictor of RAO^5^. In an attempt to decrease the diameter of the radial access hardware without sacrificing available inner lumen diameter, a sheathless approach has been suggested. Introduction of a 7 french guide catheter in a sheathless fashion, subjects the radial artery to a puncture equivalent to that created by introduction of a 5 french sheath^6^. This provides an attractive opportunity to perform procedures using adequate guide catheter lumen diameter, with a “sheath to artery ratio” that seems favorable, and poised to reduce the risk of RAO. This approach has been found to be feasible using a wide array of catheter sizes including 5 and 6 french catheters^7^. Systematic comparison of incidence of RAO using sheathless and the traditional technique using an introducer sheath has not been reported.

The objective of this study was to evaluate the incidence of RAO using sheathless technique, and compare it to the traditional approach.

## Materials and Methods

The study protocol was approved by the Institutional review board (IRB) of the Wright Center for Graduate Medical Education, Scranton, PA. The need for an informed consent was waived by the IRB board considering the retrospective nature of the analysis. A total of 1520 patients undergoing PCI performed by 1 operator, using 6 french guide catheters between from January 2008 to December 2012 at 2 tertiary care centers in Scranton, PA were studied. Patients presenting with ST-segment elevation myocardial infarction (n = 180), previous ipsilateral radial artery access (n = 43), those treated with warfarin or novel oral anticoagulant agents (n = 21), those receiving Bivalirudin as the antithrombin agent for PCI (n = 24), and those receiving ipsilateral radial arterial line insertion before completion of follow-up (n = 1) were excluded from the cohort. 161 patients underwent PCI using sheathless technique (SLG) and 1090 patients undergoing TRA PCI using the traditional technique using a sheath during (SG) were included in the analysis. Data were retrospectively collected and analysed. All patients underwent PCI procedures using a 6 french guide catheters. 6 french sheathless procedures were performed with an intent to lower the incidence of RAO as expected from previous observation^5^ by eliminating the larger profile 6 french sheath with a significant reduction in hardware profile and arterial rent. All patients received 70 units/kg unfractionated heparin intravenously after obtaining access to the radial artery lumen with a guide wire.

### Sheathless technique

After routine preparation and sterile draping, the skin of the lateral aspect of the forearm was infiltrated with 1–2 ml of preservative free lidocaine, 2–3 fingerbreaths above the styloid process. Radial pulse was palpated at that site and radial artery access was obtained using a 20-gauge teflon sheathed needle using counterpuncture technique^8^. A micropuncture kit was used, with a 0.018” nitinol mandril guide wire was placed once continuous blood flow was visualized. A “telescoped composite” introducer from the micropuncture kit, with a 0.018” guidewire capable inner catheter, and 0.035” capable outer catheter was introduced over the 0.018” guidewire. After introducing the composite into the radial artery lumen, the inner catheter was removed, vasodilators including 5 mg of Diltiazem and 200 mcg nitroglycerin were administered intraarterially through the outer catheter and a 0.035” J-tipped guide wire was advanced into the subclavian artery, using fluoroscopy if needed. A 2–3 mm dermotomy was made using a number 11 scalpel blade. The puncture site was dilated using a 6 french dilator. The guide catheter was introduced using 2 techniques.

#### Technique 1

A 5 french, 125 cm multipurpose catheter was placed inside a 6 french guide catheter (with an outer diameter of 2.08 mm) and the composite was placed over the 0.035” guidewire. The system was advanced through the puncture-site and cork-screw torque was applied if needed. The inner 5 french catheter was removed once ascending aorta was accessed. This technique has been previously described^6^.

#### Technique 2

Radial artery was punctured usin micropuncture kit using the same technique as described above. The 0.035” guidewire was removed after placing the 6 french dilator in the radial artery, and a 0.014” guide wire was placed in the subclavian artery. A 2.0/15 mm semicompliant balloon was placed in the 6 french guide catheter, and the 0.014” guidewire was placed through the balloon wire lumen. The balloon was inflated using a standard baloon inflation device at 6–8 atmospheres, the system was advanced through the puncture-site and cork-screw movement was applied if needed. The balloon and 0.014” guidewire were removed once ascending aorta was accessed as previously described^9^. All guide catheters used were non-hydrophilic with an outer diameter of 2.06 mm.

### Traditional technique

After routine preparation and sterile draping, the skin of the distal flexor aspect of the forearm was infiltrated with 1–2 ml of preservative free 1% lidocaine, 2–3 fingerbreaths above the styloid process. Radial pulse was palpated at that site and radial artery access was obtained using a 20-gauge teflon sheathed needle after which a 0.021′′ guide wire was placed through the teflon cannula into the radial artery lumen, once continuous blood flow was visualized. A 6 french hydrophilic introducer sheath (Radiofocus, Terumo Medical) with outer diameter of 2.65 mm was placed over the guide wire into the radial artery. We did not have any patients in our dataset who had their sheath size upsized during the procedure. 5 mg of Diltiazem and 200 mcg of nitroglycerin were administered via the introducer sheath in an intraarterial fashion. A 0.035′′ J-tipped guidewire was placed in the ascending aorta and a 6 french guide catheter was advanced over the wire.

All patients received aspirin 81 mg orally, and a thienopyridine agent before the procedure. The PCI procedure was completed successfully in 1251 patients. 1 patient developed coronary artery perforation, neccessitating emergent coronary artery bypass graft surgery, and was excluded from the analysis, in view of receiving an ipsilateral radial arterial line catheter for hemodynamic monitoring.

### Hemostasis technique

An inflatable band (TR band, Terumo Medical) was applied at the radial access site and the velcro straps were connected. In patients without a sheath, after removal of balloon catheter, a 0.035” J-tipped guidewire was advanced into the guide catheter and the catheter was removed over the guidewire. After removing the catheter-wire composite, the band was rapidly inflated to obtain hemostasis. Patent hemostasis was attempted in all patients, using the technique described earlier^10^. In patients with introducer sheath, the catheter was removed over the guidewire as described, inflatable band was applied at the access site, the sheath was removed after which the band was inflated to obtain hemostasis. Status of radial artery flow during hemostatic compression was available only at the time of onset of hemostasis.

Demographic and procedural data were collected in all patients. Radial artery patency was evaluated using reverse Barbeau’s test, at the time of removal of hemostatic compression band, 24 hours and 30 days after the procedure. In patients with lack of radial patency, ultrasonography was performed to evaluate patency. RAO observed at 24 hours follow-up was defined as “acute RAO”, and RAO observed at 30-day follow-up was defined as “persistent RAO”.

### Statistical analysis

Data was expressed in number (percentage) for categorical variables, and as mean ± standard deviation for continuous variables (if normally distributed) or as median ± inter-quartile range (if non-normally distributed). The total study cohort was categorized into SG and SLG. Continuous variables were compared between these 2 groups using student ‘t’ test (if normally distributed) or the Wilcoxon rank-sum test (if non-normally distributed). Categorical variables were compared using chi-squared test or Fischer’s exact test as appropriate.

In view of our event of interest (i.e. radial artery occlusion) being rare (3%) (Table 1) and the possibility of bias due to confounding, we used propensity score matching in an attempt to control for these biases. Propensity score, in our study, denotes the probability that a patient will receive the intervention of interest (use of sheath during radial access) rather than control (sheath-less radial access) with given prognostic and co-morbid variables. This is a commonly used statistical technique in observational studies wherein the groups that are compared are weighted and matched in such a way that common measured comorbid conditions and study variables are balanced between groups. Hence, after propensity matching, the resulting variability in study outcomes between groups may be inferred to be the true effect of the intervention under question and not due to confounding by other variables. Propensity scores were calculated using logistic regression model with use of sheath during radial access as the dependent dichotomous variable and factors that can potentially influence the choice of access namely; age, gender, BMI, creatinine, diabetes diagnosis, hypertension diagnosis, congestive heart failure diagnosis, tobacco use, plavix use, and fluro-time (a potential surrogate for complexity of the procedure) as dependent variables. From the distribution of propensity scores, the sheath-less group and with sheath group were categorized and caliper matched (caliper distance 0.01 points of propensity score) into 5 strata with strata 1 having the lowest likelihood of being assigned to SG and strata 5 having the highest likelihood of being assigned to the SG. Caliper matching ensures that the comorbid characteristics are tighly balanced between groups that are compared.

**Table 1:** Baseline characteristics and follow-up details of the study cohort

Variables	Total cohort	Unadjusted		P value	Propensity matched		P value
	(n = 1251)	SLG	SG		SLG	SG	
		n (%):161(13)	n (%):1090(87)		n (%): 152(12)	n (%): 1090(88)	
Age (years)	65.0 ± 12.2	64 [19]	65 [19]	0.294	64.5 [20]	65 [19]	0.413
Males- n (%)	793 (63)	96 (59.6)	697 (63.9)	0.294	91 (59.9)	697 (63.9)	0.325
BMI (Kg/m^2^)	30.4 ± 6.8	30.2 [7.7]	29.2 [8.0]	0.117	30.4 [7.9]	29.2 [7.9]	0.081
Creatinine (mg/dl)	1 [0.4]	1[0.2]	1[0.4]	0.445	1 [0.2]	1 [0.4]	0.560
Diabetes-n (%)	462 (37)	65 (40.4)	397 (36.4)	0.337	60 (39.5)	397 (36.4)	0.474
HTN–n (%)	946 (76)	125 (77.6)	821 (75.3)	0.557	118 (77.6)	821 (75.3)	0.614
Tobacco- n (%)	727 (58)	111 (68.9)	616 (56.5)	0.003	102 (67.1)	616 (56.5)	0.014
Clopidogrel-n (%)	877 (70)	101 (62.7)	776 (71.2)	0.034	99 (65.1)	776 (71.2)	0.130
Heparin during procedure (units)	9000 ± 3000	9000 ± 3000	8500 ± 3000	0.899	9000 ± 3000	9000 ± 3000	0.999
Time until TR band removal (minutues)	200 ± 30	200 ± 40	200 ± 30	0.718	200 ± 30	200 ± 40	0.913
Fluoro time (min)	5.4 [7]	5.7 [7.2]	5.3 [6.9]	0.910	5.7 [7.2]	5.3 [6.9]	0.838
Patent Hemostasis	978 (78)	127 (78.8)	851 (78.1)	0.919	118 (77.6)	851 (78.1)	0.917

SG: Group with Sheath, SLG: Sheathless Group.

The association between RAO at 3 time points (independent variables) namely; (1) after removal of hemostatic compression band, (2) 24 hours after the procedures, and (3) 30 days after the procedure, with use or non-use of sheath during radial access (dependent variable) was assessed using 3 logistic regression models; model 1: uni-variate (unadjusted), model 2: multi-variate (adjusted for age, gender, BMI, hypertension diagnosis, tobacco use, plavix use, diabetes diagnosis and patent hemostasis) and model 3 (propensity matched).

Recanalization rates (%) at 24 hrs (acute recanalization) and 30 days post procedure (sub-acute recanalization) were calculated and compared between the two groups (SG Vs SLG). Uni-variate, muti-variate (adjusted for age, gender, diabetes; variables selected by the process of forward selection) logistic regression analyses were performed to assess the association between re-canalization at 24 hours and use or non-use of sheath during radial access. A similar adjusted analysis could not be performed for 30 day re-canalization analysis due to small numbers (n = 31) who achieved re-canalization at 30 days. P < 0.05 was considered statistically significant for these analyses. All Data analyses were performed using STATA 11 statistical software.

## Results

1251 patients formed the study cohort. Of these, 161 (13%) were in the SLG (99 patients underwent sheathless insertion using technique 1, and 62 patients underwent sheathless guide catheter insertion using technique 2) and 1090 (87%) were in the SG. Baseline characteristics of the study cohort, the SLG and SG, pre and post propensity matching, are detailed in Table 1.

**Figure 1:**
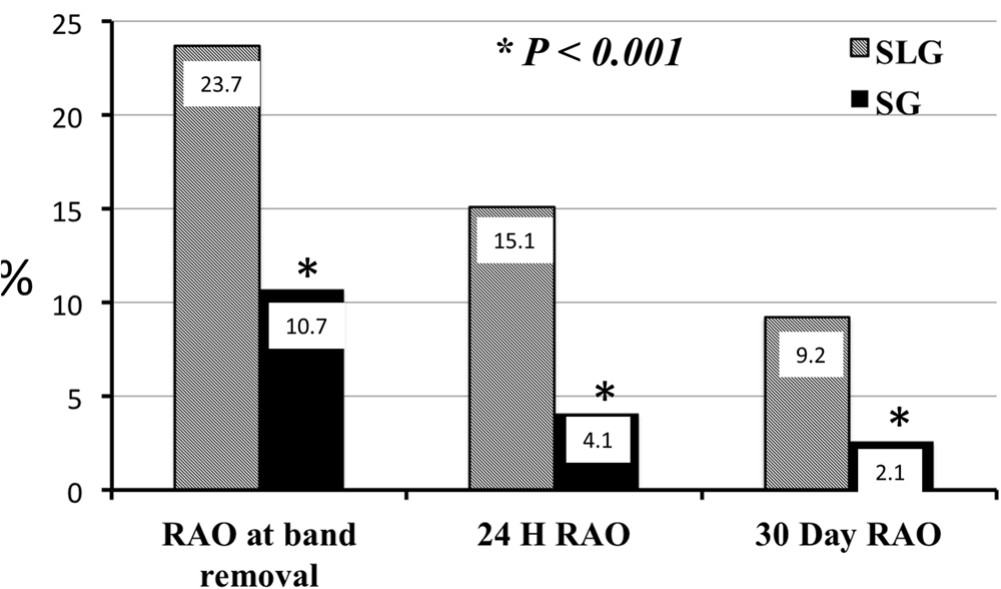
Incidence of radial artery occlusion during follow-up. Incidence of radial artery occlusion at band removal, 24 hours and 30 days post PCI when SLG was compared with SL group in the propensity matched analysis.

**Figure 2:**
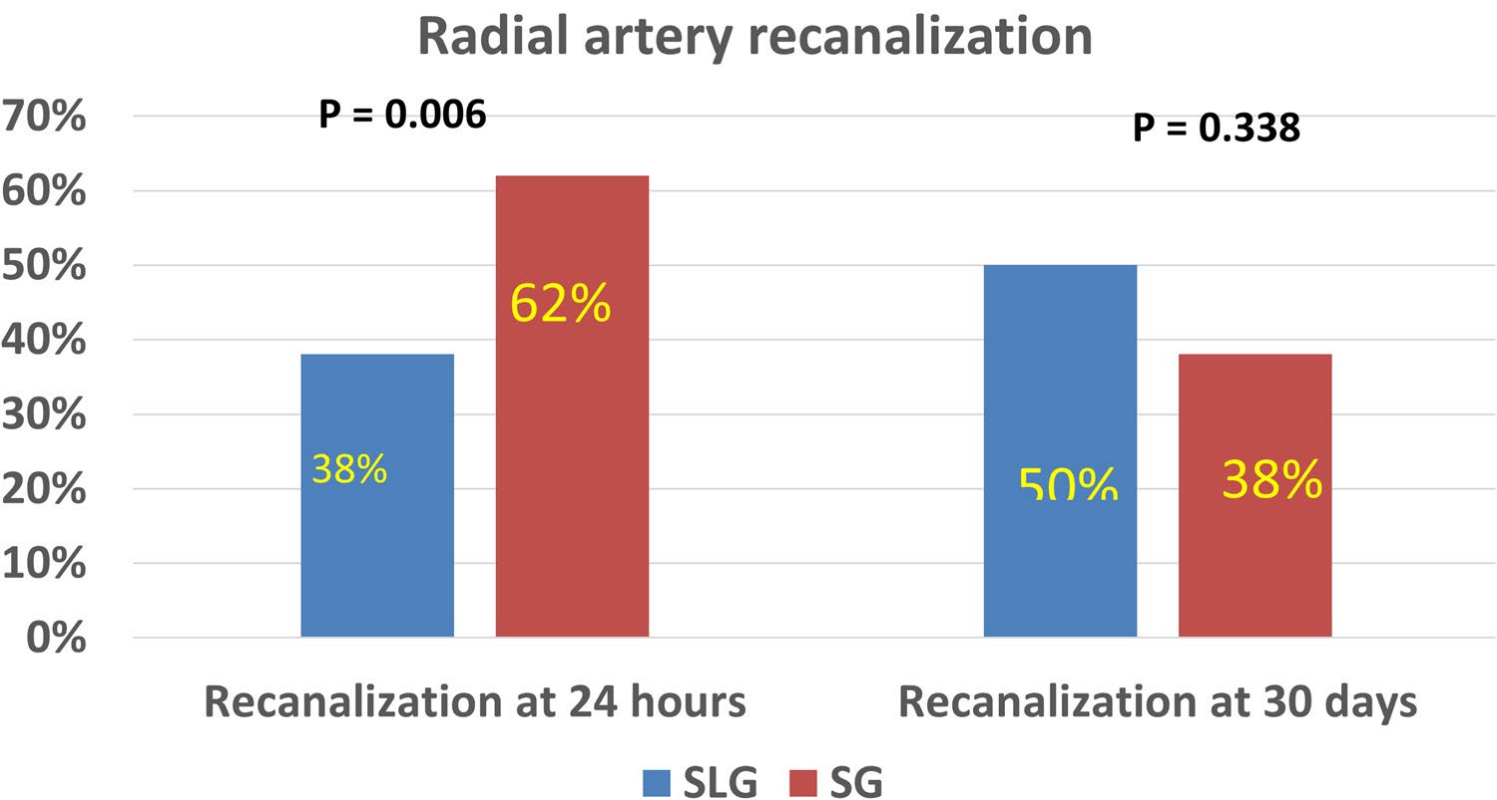
Incidence of radial artery recanalization during follow-up. Incidence of radial artery recanalization at 24 hours and 30 days post PCI when SLG was compared with SL group in the propensity matched analysis.

Of the 1251 study cohort participants, 162 (13%), 73 (6%) and 43 (3%) participants were noted to have RAO at the time of removal of hemostatic compression band, 24 hours and 30 days post procedure respectively. Among the 161 SLG participants, 45 (28%), 28 (17%) and 15 (9%) patients were noted to have RAO at pre-discharge, 24 hours and 30 days post procedure respectively. In the 1090 SG participants, 117 (11%), 45 (4%) and 28 (3%) respectively were noted to have RAO at the time of removal of hemostatic compression band, 24 hours and 30 days post procedure. The SLG experienced higher incidence of radial artery occlusion at band removal (P < 0.001), 24 hours (P < 0.001) and 30 days post procedure (P < 0.001) follow-up. Further, use of sheath during radial access was protective against RAO when compared to a sheath less approach in the unadjusted, adjusted and propensity matched analyses for band removal, 24 hours and 30 day RAOs (Tables 2–4 and Fig. 1). Among patients who had RAO, the SG had higher rate of radial artery recanalization at 24 hours compared to SLG (P = 0.006). There was no significant difference in recanalization rate between the two groups at 30 days (Fig. 2). Ultrasound data were available on all 42 patients with RAO at 30-day follow-up. The radial artery diameter in patients with 30 day RAO in SG was 1.97 ± 0.2 mm, compared to 2.0 ± 0.2 mm in SLG (P = 0.2).

**Table 2:** Association between sheath less radial access and radial artery occlusion at band removal.

Variables	Univariate analysis	Multi-variate analysis		Propensity matched analysis		
	OR (95% CI)	P value	OR (95% CI)	P value	OR (95% CI)	P value
Age	1.00 (0.99–1.01)	0.895	0.99 (0.98–1.02)	0.954	—	—
Females	1.05 (0.75–1.48)	0.768	0.82 (0.53–1.29)	0.393	—	—
BMI	0.99 (0.97–1.02)	0.494	0.98 (0.94–1.01)	0.147	—	—
Creatinine	0.97 (0.81–1.16)	0.763	—	—	—	—
Diabetes	1.07 (0.76–1.50)	0.705	0.94 (0.59–1.50)	0.811	—	—
Hypertension	1.02 (0.69–1.49)	0.922	1.22 (0.71–2.09)	0.470	—	—
Tobacco use	1.11 (0.79–1.57)	0.511	1.08 (0.69–1.68)	0.725	—	—
Plavix use	0.95 (0.66–1.36)	0.773	1.02 (0.64–1.64)	0.923	—	—
Flurotime	0.99 (0.97–1.02)	0.613	—	—	—	—
Patent Hemostasis	0.03 (0.02–0.04)	<0.001	0.02 (0.01–0.03)	0.001	—	—
Sheath use	0.31 (0.21–0.46)	<0.001	0.1 (0.05–0.190)	<0.001	0.20 (0.13–0.32)	< 0.001

**Table 3:** Association between sheath less radial access and radial artery occlusion at 24 hours.

Variables	Univariate analysis	Multi-variate analysis		Propensity matched analysis		
	OR (95% CI)	P value	OR (95% CI)	P value	OR (95% CI)	P value
Age	1.00 (0.98–1.02)	0.941	0.99 (0.97–1.02)	0.918	—	—
Females	1.85 (1.15–2.97)	0.011	1.62 (0.92–2.85)	0.093	—	—
BMI	0.99 (0.95–1.02)	0.444	0.97 (0.93–1.01)	0.177	—	—
Creatinine	0.99 (0.91–1.07)	0.760	—	—	—	—
Diabetes	1.82 (1.13–2.92)	0.013	1.86 (1.03–3.36)	0.040	—	—
Hypertension	0.91 (0.53–1.56)	0.736	1.01 (0.50–2.04)	0.974	—	—
Tobacco use	1.41 (0.86–2.32)	0.175	1.44 (0.79–2.61)	0.227	—	—
Plavix use	0.99 (0.59–1.65)	0.963	0.96 (0.51–1.80)	0.892	—	—
Flurotime	0.99 (0.96–1.03)	0.746	—	—	—	—
Patent Hemostasis	0.03 (0.02–0.07)	<0.001	0.03 (0.01–0.05)	<0.001	—	—
Sheath use	0.20 (0.12–0.34)	<0.001	0.12 (0.06–0.24)	<0.001	0.13 (0.07– 0.25)	<0.001

**Table 4:** Association between sheath less radial access and radial artery occlusion at 30 days.

Variables	Univariate analysis	Multi-variate analysis		Propensity matched analysis		
	OR (95% CI)	P value	OR (95% CI)	P value	OR (95% CI)	P value
Age	0.99 (0.97–1.02)	0.983	0.99 (0.97–1.03)	0.896	—	—
Females	1.95 (1.05–3.61)	0.034	1.55 (0.77–3.15)	0.222	—	—
BMI	0.98 (0.94–1.03)	0.528	0.98 (0.93–1.02)	0.320	—	—
Creatinine	0.99 (0.94–1.05)	0.819	—	—	—	—
Diabetes	2.12 (1.14–3.94)	0.017	2.39 (1.14–5.05)	0.021	—	—
Hypertension	0.79 (0.40–1.58)	0.521	0.79 (0.34–1.87)	0.597	—	—
Tobacco use	1.46 (0.76–2.79)	0.256	1.64 (0.78–3.44)	0.194	—	—
Plavix use	0.85 (0.44–1.62)	0.621	0.80 (0.37–1.76)	0.586	—	—
Flurotime	0.99 (0.95–1.04)	0.799	—	—	—	—
Patent Hemostasis	0.01 (0.001–0.04)	<0.001	0.01 (0.001–0.04)	< 0.001	—	—
Sheath use	0.28 (0.14–0.54)	<0.001	0.22 (0.10–0.50)	< 0.001	0.18 (0.10–0.40)	< 0.001

Among the 162 participants from the study cohort who were noted to have RAO at band removal, 89 re-canalized (55%) within 24 hours (acute recanalization). Of these, 17 (38%) and 72 (62%) were noted in the SLG and SG respectively. SG experienced significantly higher incidence of acute recanalization when compared to the SLG in the chi-squared analysis (P = 0.006). This was confirmed in the unadjusted (OR: 2.5, 95% CI: 1.3–5.4, P = 0.007) and adjusted (OR: 2.4, 95% CI: 1.11–5.01, P = 0.025) logistic regression analyses. At 30 days follow-up, 31 more study cohort participants were noted to have recanalized [14 (50%) in the SLG and 17 (38%) in the SG but the 2 groups were similar (P = 0.338) with regards to the rates of recanalization at this time point.

## Discussion

Our data show that the incidence of RAO using a sheathless technique is significantly higher, compared to the traditional technique using a hydrophilic introducer sheath. This is counterintuitive as the sheathless technique provides a smaller “catheter to artery ratio” compared to the introducer sheath, which is typically approximately 2 french sizes larger than a corresponding sized catheter^6^. The most likely reason for this unexpected observation, is the higher intimal trauma at the arterial entry site associated with sheathless catheter insertion. During the process of inserting the catheter into the radial artery lumen without a sheath, a composite of two telescoped catheters, a dilator-catheter or balloon-catheter composite are advanced through the skin into the arterial lumen over a guidewire. In view of large step-like transitions, especially with the telescoped catheter composite, the potential for a large “razor-effect” exists^11^. In the absence of spasm or variant anatomy, radial artery puncture site is the point of maximal resistance along the path of catheter transit. Using a sheath based approach, the length of interaction between this high resistance entry point and introducer sheath, is usually 4–16 centimeters, depending on the length of the introducer sheath. A hydrophilically coated introducer sheath significantly decreases the amount of friction, compared to a non-hydrophilic sheath, both during insertion and removal^12^, hence further reducing intimal trauma.

In contrast, a sheathless approach using the traditional guide catheters, requires the advancement of the catheter through the puncture site, with the length of interaction of the order of 70–90 cm. This large increase in the length and duration of friction, with absence of hydrophilic coating on majority of the available guide catheters, sizeably increases the likelihood and severity of intimal abrasion and trauma associated with the insertion and removal of a catheter using sheathless technique. This increase in intimal trauma, might be a reason for the observed increase in the incidence of RAO compared to the larger diameter but less abrasive sheath based approach. The length related friction and associated larger insult is expected to be a persistent issue, even when hydrophilic-coated sheathless guide catheters are used. The impact of reduction in friction as a result of hydrophilicity of newer guide catheters, on RAO will need to be re-evaluated.

The lower rates of acute recanalization in the patients with RAO in sheathless cohort compared to those who received an introducer sheath, likely are a result of an unfavorable balance of pro-coagulant and thrombolytic processes in the local mileu, as well as more prolonged healing process, as a result of the larger burden of index injury. These observations emphasize the importance of the different determinants of hardware related vessel injury, and corroborate its multifactorial construct.

RAO and its clinical significance become more relevant as more complex patient subsets are treated using transradial access, with an increasing need for repeat procedures in view of staging as well as progression and/or restenosis. Further evaluation of RAO using these two techniques in a randomized fashion or a larger registry based dataset with multiple operators and centers will further validate our findings.

### Limitations

The retrospective design of this evaluation exposes it to the limitations inherent to the design, although propensity matching makes these results incur the least confounding from identifiable covariates. The small sample size of the SLG group raises the possibility of immigrative selection bias; operator choice depending on patient characteristics is possible. Our dataset is restricted to use of non-hydrophilic guide catheters, and hence our results are not generalizable to hydrophilic guide catheters.

## Conclusion

Transradial PCI using “sheathless” technique is associated with a higher incidence of RAO compared to the technique using hydrophilic introducer sheath. Recanalization is less likely once RAO occurs, in the “sheathless” cohort compared to the hydrophilic sheath technique^13–16^.

### Impact on daily practice

1. Post procedural loss of patency is the most common structural complication of TRA with incidence varying from 2–10%.
2. Our study shows that Transradial PCI using “sheathless” technique is associated with a higher incidence of RAO compared to the technique using hydrophilic introducer sheath. Recanalization is less likely once RAO occurs, in the “sheathless” cohort compared to the hydrophilic sheath technique.
3. Our results need validation in randomized trials.
